# Laterally Excited Resonators Based on Single-Crystalline LiTaO_3_ Thin Film for High-Frequency Applications

**DOI:** 10.3390/mi15121416

**Published:** 2024-11-26

**Authors:** Chongrui Guan, Xingli He

**Affiliations:** School of Electronic and Information Engineering, Soochow University, Suzhou 215006, China; 20225228088@stu.suda.edu.cn

**Keywords:** LiTaO_3_ film, lamb mode, Laterally excited bulk resonators, filter

## Abstract

High-performance acoustic resonators based on single-crystalline piezoelectric thin films have great potential in wireless communication applications. This paper presents the modeling, fabrication, and characterization of laterally excited bulk resonators (XBARs) utilizing the suspended ultra-thin (~420 nm) LiTaO_3_ (LT, with 42° YX-cut) film. The finite element analysis (FEA) was performed to model the LT-based XBARs precisely and to gain further insight into the physical behaviors of the acoustic waves and the loss mechanisms. In addition, the temperature response of the devices was numerically calculated, showing relatively low temperature coefficients of frequency (*TCF*) of ~−38 ppm/K for the primary resonant mode. The LT-based XBARs were fabricated and characterized, which presents a multi-resonant mode over a wide frequency range (0.1~10 GHz). For the primary resonance around 4.1 GHz, the fabricated devices exhibited a high-quality factor (Bode-Q) ~ 600 and piezoelectric coupling (*k_t_*^2^) ~ 2.84%, while the higher-harmonic showed a greater value of *k_t_*^2^ ~ 3.49%. To lower the resonant frequency of the resonator, the thin SiO_2_ film (~20 nm) was sputtered on the suspended device, which created a frequency offset between the series and shunt resonators. Finally, a ladder-type narrow band filter employing five XBARs was developed and characterized. This work effectively demonstrates the performance and application potential of micro-acoustic resonators employing high-quality LT films.

## 1. Introduction

In the 5G era, the importance of micro-acoustic filters in the RF front-end module is ever-increasing. Over the past decade, filters based on surface acoustic wave (SAW) devices and film bulk acoustic wave resonators (FBAR) have experienced tremendous market growth globally [1,2]. However, the allocation of the new frequency bands has imposed challenges for the mainstream bulk LiNbO_3_ (LN)/LiTaO_3_ (LT)-based SAW (<2.5 GHz) and the established AlN-FBAR (<6 GHz) techniques.

A few new techniques have been developed to improve the performance of the acoustics resonators, e.g., the high-performance Lamb wave resonator based on the AlN/diamond-based composites operating at the microwave frequency band have shown great potential in wireless communications [3,4,5]. The benefits of the piezoelectric composite employing the AlN and diamond have been systematically analyzed, and new resonator prototypes, such as the bulk transducers with double side metallic topology, have been investigated, which gives the largest piezoelectric coupling *k_t_*^2^ up to 4.5% with a super high frequency up to 12 GHz [6]. The reported works show the great promise of such new composite structures for high-performance acoustic filter development. On the other hand, in recent years, acoustic wave devices based on single-crystalline piezoelectric (PE) thin films, such as LN and LT, have attracted significant attention. One technique that has enabled the fabrication of single-crystalline piezoelectric thin films (with thickness ~μm) is crystal-ion-slicing (CIS). For the CIS process, firstly, an ion (H^+^ or He^+^) implantation is performed, and the depth of the defect layer can be defined by adjusting the ion energy and flow. Prior to direct wafer bonding, the LN or LT wafer is covered by a dielectric (typically SiO_2_) layer on the implanted side. Then, the bonding surfaces are cleaned and prepared to exhibit hydrophilic properties and the LN/LT substrate is bonded with a silicon holding wafer. After that, the bonded pairs were annealed until the LN/LT thin film layer is transferred. Finally, the chemical mechanical polishing (CMP) process is performed to smooth the piezoelectric thin film surface, and the hybrid composite substrate with single-crystalline LN/LT layer can be obtained; more detail can be found in refs [7,8]. By employing high-quality materials obtained through CIS, acoustic resonators with exceptional performance can be developed [9,10,11,12,13,14,15]. Compared with the devices based on LT films, the acoustic resonators utilizing the LN films, generally speaking, exhibited a higher effective electromechanical coupling coefficient (*k_t_*^2^) and phase velocity (*V_p_*). These features make the single-crystalline LN film attractive for developing wideband and high-frequency filters [16,17]. However, the LN thin film has its intrinsic limitation concerning the temperature response; the relatively high temperature coefficient of frequency (*TCF*) will significantly reduce the reliability of the devices, especially in high-power scenarios. Additionally, it is crucial to address the issue of cost in the production of single-crystalline LN thin films, as it could hamper commercial applications. In contrast, single-crystalline LT films feature a low-temperature coefficient and are more cost-effective. They have the potential to serve as a strong competitor and potential substitute for the thin LN film for high-performance electronics. A few works concerning acoustic wave resonators based on high-quality LT thin films have been reported. V. Plessky and collaborators conducted numerical calculations on the acoustic radiation characteristics for LSAW resonators based on a thin LT plate [18,19]. Yan et al. reported the details about the wafer-scale bonding process to form an LT on an insulator (LTOI) substrate and obtained a SAW resonator with the Rayleigh mode frequency of 347 MHz [7]. L. Zhang et al. reported high-performance acoustic wave devices developed on LiTaO_3_/SiC hetero-substrates, demonstrating scalable resonances ranging from 1.19 to 4.91 GHz and with high quality (*Q*) values between 900 and 8100 [12]. Besides the SAW devices with solidly mounted structures, the suspended-type resonators were also explored. For example, N. Assila and co-workers developed a high-frequency resonator with anti-symmetric (*A*_1_) Lamb wave mode by utilizing a LiTaO_3_ plate [11]; Y. Xue et al. reported high *Q* (~1690) XBARs using an LT/SiO_2_ (670 nm/1500 nm) composite plate, which resonated at ~2.46 GHz [20]. Through these pioneering works, we have gained considerable knowledge about LT thin film-based resonators, yet further work is required to promote this technique for applications, e.g., the precise modeling of these devices.

To gain an in-depth understanding of the LT thin film-based micro-acoustic resonators, a complete three-dimensional (3D) XBAR model was developed and analyzed using finite element analysis (FEA, COMSOL Multiphysics 6.1). This approach allowed for the characterization of various aspects of these devices, including their vibration mode, loss mechanism, and temperature characteristics. To verify the accuracy of the simulations, LT thin film-based XBARs were fabricated on a 420 nm LT plate. Nanoscale interdigitated transducers (IDTs) with a narrow finger width of ~500 nm and a large period of 5.9 μm were formed on the piezoelectric plate, which presented a primary resonance ~4.1 GHz, and the fabricated devices had a coupling factor *k_t_*^2^ ~2.84% for this resonant mode. One practical application of these LT thin film-based XBARs was demonstrated through the preparation of a ladder-type bandpass filter. Overall, this research provides a comprehensive understanding of LT thin film-based micro-acoustic resonators, showcasing their potential for high-frequency applications.

## 2. Device Fabrication

**Figure 1:**
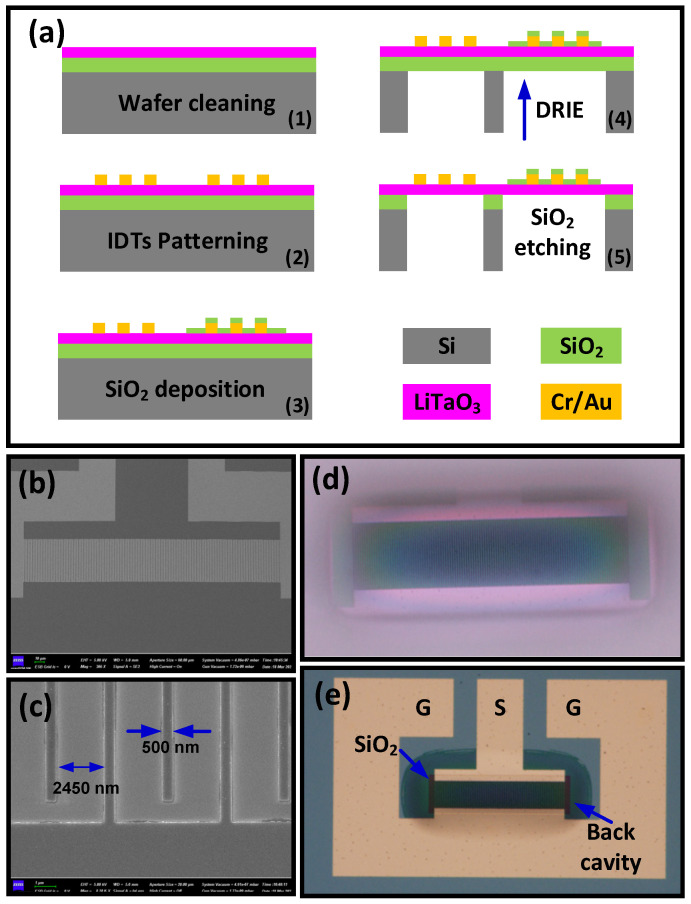
(**a**) Fabrication process flow of LT-based XBARs; (**b**) SEM image of the resonator before etching the back trench; (**c**) zoom-in image showing the width of finger pairs; microscope images of the device with a back view (**d**) and a top view (**e**).

The proposed resonators were implemented on an ion-sliced composite wafer with a 42° YX-cut single-crystalline LT layer (~420 nm) bonded on a SiO_2_/Si (2 μm/525 μm) carrier wafer. The wafer was prepared by NanoLN (Jinan Jingzheng Electronics Company Ltd., Jinan, China) and is commercially available; Figure 1a shows the fabrication process. According to simulations, XBARs with a finger width of 500 nm and feature wavelength (λ) of 5900 nm were patterned using an electron beam lithography (EBL) system (JEOL-9500FS, JEOL, Tokyo, Japan) and lift-off process, with the Cr/Au electrode thickness being 20 nm/50 nm. After patterning the IDTs, a thin layer of SiO_2_ (~20 nm) was partially deposited on the surface, which is used to create a frequency offset between resonators. The carrier Si substrate was released in the DRIE process, and the suspended membranes with clamped boundaries were obtained. The remaining SiO_2_ layer was removed by the buffered oxide etchant, and finally, the LT-thin film based XBARs were prepared. Figure 1b shows the SEM image of the patterned IDTs before etching the back trench. The zoom-in image shown in Figure 1c presents the details of the electrodes; a finger width of 500 nm can be obtained, with no residual metal particles existing between electrodes. The backside view of the device after the backside Si release is shown in Figure 1d; a semi-transparent suspended structure with IDTs can be observed. The obtained resonator structure is shown in Figure 1e. The gray area with a curved boundary denotes the released cavity, and the dark rectangle on top of the IDTs is the SiO_2_ mass-loading layer. The active area is fully suspended without substantial bending or cracking. The electrical characteristics of the fabricated resonators were measured using a Keysight P9375A network analyzer (Keysight, Santa Rosa, CA, USA) connected with the coplanar ground-signal-ground (GSG) probes. The performance of the devices will be analyzed in Section 4.

## 3. Numerical Simulations

The accurate modeling of the LT thin film-based micro-acoustic resonator is crucial for gaining a deeper understanding of the characteristics of the devices and in optimizing the design. The FEA method is widely used for simulating piezoelectric devices, which serves to solve the constitutive piezoelectric equations and facilitate the modeling of the micro-acoustic resonator with assorted structures. This work uses the FEA tool (COMSOL Multiphysics) to investigate the characteristics of the LT thin film-based XBARs.

Indeed, in much of the existing literature on the FEA modeling of piezoelectric resonators that incorporate the IDT structure, a classical simplified two-dimensional model is commonly used. This model can be typically characterized by a piezoelectric substrate and one pair of fingers, while the periodic boundary condition is applied on the periphery of the piezoelectric substrate. This simplified model offers high efficiency in gaining a basic view of the devices and allows for extracting valuable information such as the resonant frequency, mechanical coupling coefficient, and vibration mode shapes. However, it is important to note that an actual acoustic resonator is not infinite in size. For a suspended resonator, various factors, such as the aperture width, period number, gratings, gap between the IDTs and the bus line, the reflections from the clamped boundaries, etc., will impact the performance of these devices. Also, the simplified 2D model may not capture all of the critical information accurately. To overcome these drawbacks, herein, the complete 3D models have been developed; even though the 3D simulations will consume considerable computing resources, it is beneficial to gain a more comprehensive understanding of the physics behind the resonant behaviors and accurately predict the electrical characteristics.

**Figure 2:**
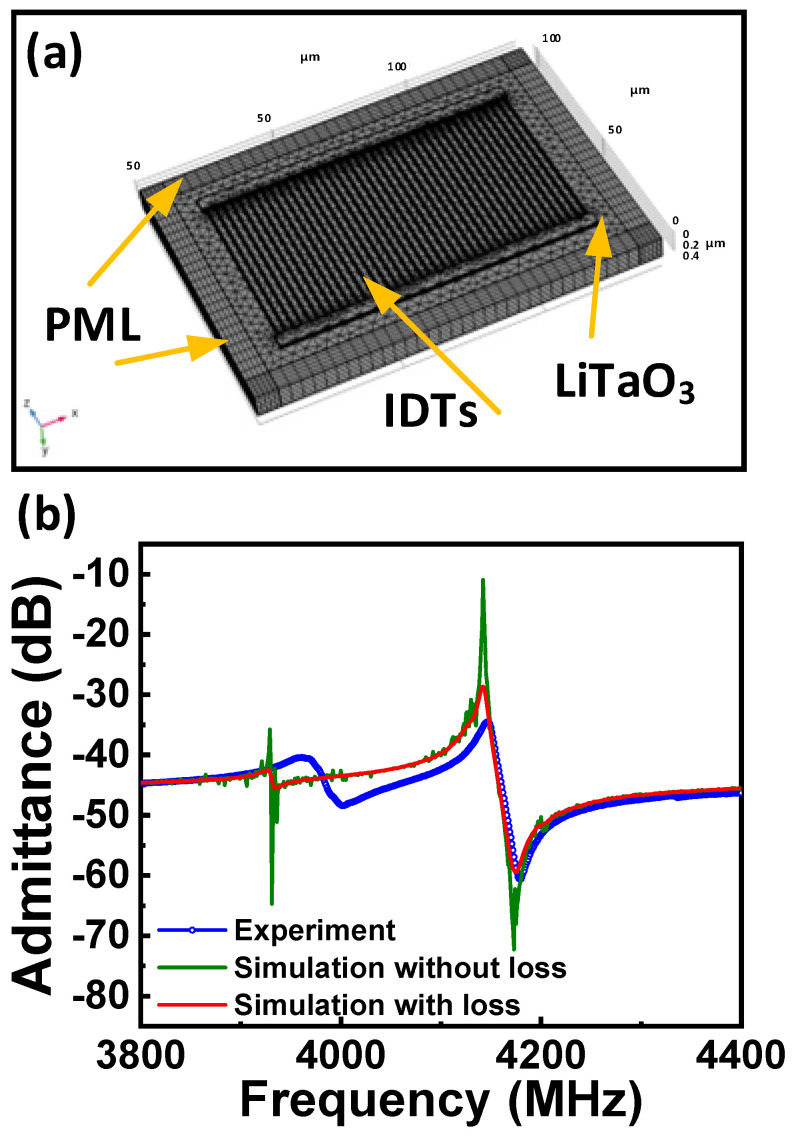
(**a**) 3D geometry of the proposed plate wave resonator using LT thin film with meshed domains, displaying the finite element distribution; the geometry scale is magnified 20 times along the thickness of the thin plate for easier discernment. (**b**) Comparison of the impedance characteristics between the accurate 3D FEM models and experimental results.

As shown in Figure 2a, the 42° YX (Euler angle (0, −48°, 0°)) LT thin film with a thickness of 420 nm was chosen as the piezoelectric plate. The IDTs with a small metalized ratio of ~0.169 were arranged to launch the acoustic wave. In the design, the feature width of the fingers is fixed at 500 nm, and the space between fingers is 2.45 μm, yielding the suspended device with a characteristic wavelength *λ* = 5.9 μm. Twenty pairs of Cr/Au (20 nm/50 nm) electrodes were designed to stimulate the resonant behavior. The acoustic aperture and gap between IDTs were set at 28 μm and 1 μm, respectively. The perfectly matching layers (PMLs) were adopted to mitigate the boundary reflections to reduce computational complexity. Rationally compartmentalizing the geometry is very important to the results of the numerical simulation. A precisely meshed XBAR model can be obtained by appropriately partitioning the surface with free triangular and quad structures, further enhanced by sweeping the geometric partitions and distribution operation. The up and down IDT electrodes were set as terminals, with alternative voltage signals (±1 V) applied. By following these design parameters, the electrical characteristics of the devices can be simulated for a comprehensive understanding of the device’s performance and resonant behavior.

Figure 2b compares the admittance spectra of the complete 3D model (the olive line) and the prepared device (the blue dotted line). A prominent resonant (*f_r_*) around 4.14 GHz can be observed, and the anti-resonant (*f_a_*) appears at ~4.17 GHz, highly consistent with experimental results. However, as the loss factors (dielectrics loss, Ohmic loss, mechanical damping, etc.) were not considered in the FEM model, the simulated result exhibits a much higher high/low impedance ratio; in addition, the peaks at *f_r_* and *f_a_* appear much sharper in the simulation than that from experiments. Besides the primary resonant, ripples are observed at ~3930 MHz in the admittance spectrum. The spurious responses are mainly attributed to the waves vibrating close to the IDT bus line, the front or back boundary of the resonant cavity. To address these discrepancies, a revised model considering the loss factors was also simulated. The obtained admittance characteristic, represented by the red curve, is much smoother than the one obtained without considering the loss factor. In this case, the high-frequency loss of the Cr and Au electrodes was considered, which is manifested by the change in the relative permittivity (*ε_r_*), and can be expressed as:

(1) $$\varepsilon_{r}=1-\frac{\sigma }{i\omega },$$

where *σ* and *ω* denote the conductance of the metal and the angular frequency, respectively. In addition, the mechanical damping and the dielectric loss of the LT thin plate were also considered in the simulation; the structural loss factor *η_s_* and the dielectric loss factor *η_εs_* are set to 0.003 and 0.002, respectively. The simulation result of the model considering these loss factors is closer to the experiment. Compared with the scenario where the loss factors were neglected, the amplitude of the primary resonance is significantly attenuated. Nevertheless, the spikes caused by the spurious modes were also diminished, leading to a smoother spectrum. Overall, incorporating these loss factors enabled the model to achieve a higher level of accuracy.

**Figure 3:**
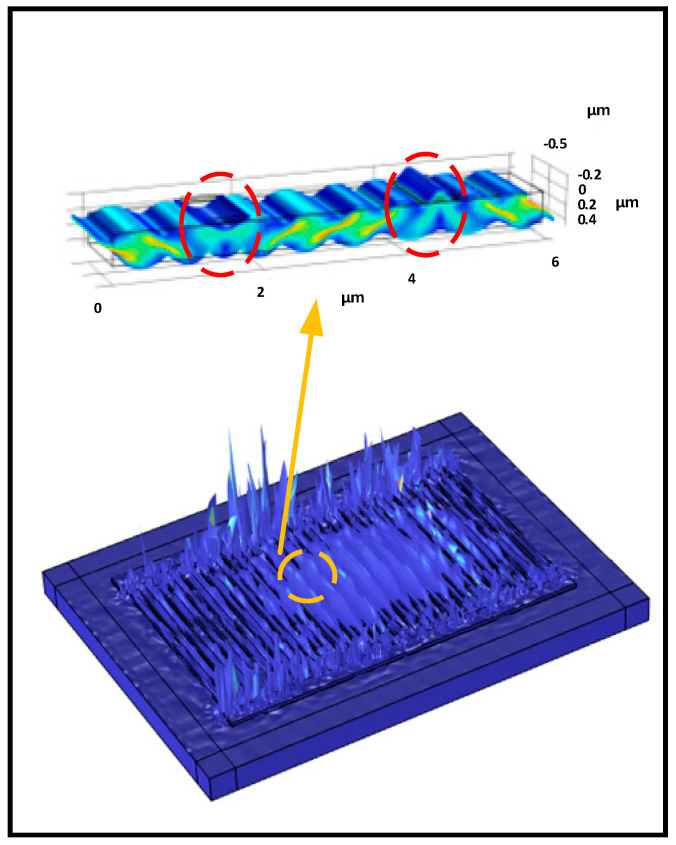
COMSOL simulation results show the displacement mode shape of the device around the primary resonance; the upper image depicts the displacement mode shape of the truncated strip comprising one finger pair.

The vibration behaviors of the primary resonant mode were evaluated, and the simulated displacement mode shape is presented in Figure 3. The deformations were concentrated at the center of the IDTs, accompanied by spikes located at the gap area between IDTs and the bus line. These spikes resulted in spurs appearing in the spectrum. To gain a clear understanding of the vibration characteristics of the wave mode, a 2.5D model representing a truncated strip of the resonator was also analyzed; the displacement mode shape is shown in the top half of Figure 3. As a plate wave device, the main resonant of XBAR, is related to the anti-symmetric mode (*A*_1_). However, as discussed in Ref. [21], the *A*_1_ description is inappropriate. The displacements beneath the metal electrodes (circled by the red dashed line in Figure 3) cannot be described as *A*_1_. Instead, it is much closer to the *A*_3_ Lamb wave. Therefore, the wave mode is a composite mode that matches the *A*_1_ field distribution in the LT plate and between finger pairs. The precise modeling of the device is beneficial to gain a deeper understanding of the physical behaviors behind the phenomenon and contributes to the optimization of the design. Although an approach to accurately modeling XBAR was introduced, there is still room for further improvement in the model, especially by considering more loss factors to achieve a higher precision.

**Figure 4:**
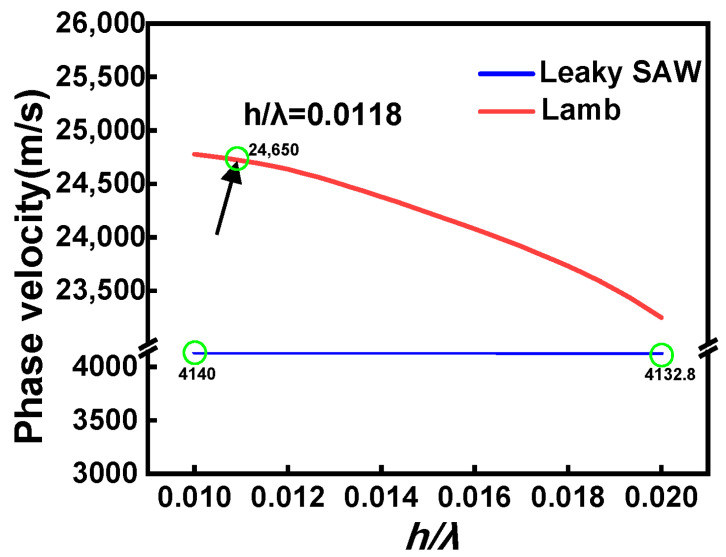
The comparison of the SAW velocities in a bulk crystal and a thin crystalline film of LiTaO_3_.

The SAW velocities in a bulk crystal and a thin crystalline film of LiTaO_3_ were compared (as shown in Figure 4). On a bulky substrate, the Leaky wave (LSAW) can be launched by the IDTs, and the phase velocity of LSAW is close to that of the surface skimming bulk acoustic wave (SSBAW), which is ~4200 m/s for the chosen crystal. Increasing the value of *h*/*λ* (a ratio between the thickness of the electrode and the device wavelength) will lead to a certain amount of decline in the LSAW velocity, while for devices based on the suspended piezoelectric thin film, the Lamb waves can be generated with electrical excitation, which presents very high phase velocity (>20,000 m/s) when the *h*/*λ* is small. In our case (*h*/*λ* = 0.0118), the obtained wave velocity for the main resonant is ~24,650 m/s, which is close to theoretical prediction and much higher than the LSAW. It should be noted that the velocity of the Lamb wave will decrease rapidly with increasing *h*/*λ*, so a thin electrode layer is preferred for high-frequency applications.

The temperature characteristics of the devices were also analyzed. The normalized material coefficients can be expressed by following polynomial expansion, using the method of least squares:

(2) $$X\left(T\right)=X_{ref}\left[1+\overset{N}{\underset{n=1}{\sum }}\alpha_{n}{\left(\Delta T\right)}^{n}\right],$$

where *X_ref_* manifests the elastic constant, piezoelectric constant, dielectric constant, and density of lithium tantalate at 25 °C, the first-order (*α*_1_) and the second-order (*α*_2_) temperature coefficients of the LT layer were considered, which can be found in Ref. [22]. The material constants of Cr and Au are listed in Table 1.

**Figure 5:**
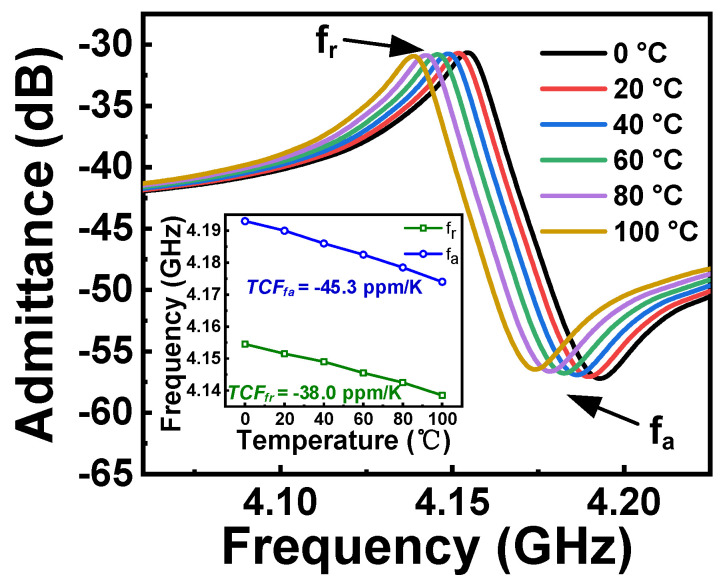
Comparison of the impedances of LT thin film-based acoustic resonators under different temperatures.

According to the findings presented in Figure 5, it can be observed that an increase in temperature leads to a decrease in resonant frequencies. The simulation results illustrate that the main resonant mode shows an approximately linear negative frequency shift as the temperature increases. The calculated temperature coefficient of the frequency (TCF) is ~38 ppm/K for the resonant frequency (*f_r_*) and ~45.3 ppm/K for the anti-resonant frequency (*f_a_*). To further reduce the TCF of the device, a thin SiO_2_ compensation layer can be used [20].

## 4. Experimental Results and Discussion

**Figure 6:**
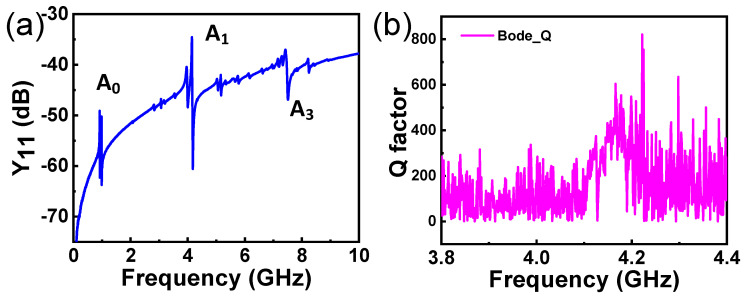
(**a**) The measured admittance (Y_11_) spectrum of the resonator over a wide frequency range. (**b**) The calculated Bode-Q of the resonator near the primary resonance.

Figure 6a exhibits the admittances (Y_11_) of a resonator based on the LT thin plate with the structural parameters in the previous section. Generally speaking, the resonator represents three prominent resonant modes over a wide frequency range (from 0.1 to 10 GHz), corresponding to the *A*_0_, *A*_1_, and *A*_3_ Lamb waves, respectively. As simulations predicted (Figure 2b), the primary resonant appears at ~4.14 GHz. The *A*_1_ mode displays an electromechanical coupling coefficient (*k_t_*^2^) of ~2.84%, while the higher harmonic (~7.4 GHz) shows a slightly higher *k_t_*^2^ of ~3.49%. Figure 6b illustrates the calculated Bode-Q of the resonator, demonstrating a *Q* value of ~600 when resonant.

The potential of the XBARs for filter application was explored. Considering the architecture of a bandpass filter, the ladder-type structure was commonly employed, which typically consisted of shunts and series resonators with frequency offset. To enhance the out-of-band rejection of the filter, the acoustic resonators typically embody tens to hundreds of IDT pairs, so XBARs with new configurations were designed. The key parameters of the series and shunt resonators composing the filter are listed in Table 2.

**Figure 7:**
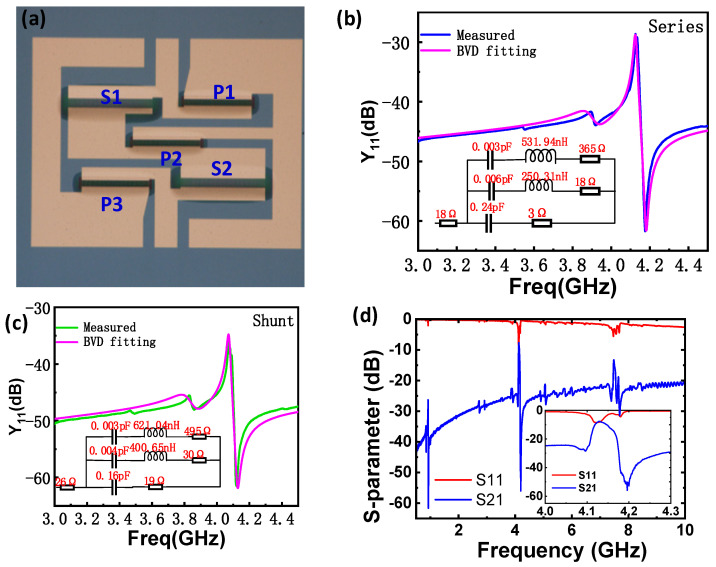
(**a**) Microscopy image of the prepared ladder-type filter; the admittance for representative one-port resonator to compose the series (**b**) and shunt (**c**) branch of the filter; (**d**) frequency response of the LT-plate-based filter over a wide frequency range, where the zoom-in images of the transmission (S_21_) and return loss (S_11_) of the filter in the vicinity of the passband was inserted.

As shown in Figure 7a, a bandpass filter consisting of five resonators was designed and fabricated. The device had a compact footprint of 1.04 mm × 0.88 mm. The filter comprised two identical series (X1, X2) and three identical shunt (X3, X4, X5) resonators. The admittances and the modified Butterworth Van-Dyke (MBVD) circuit parameters are displayed in Figure 7b,c; the resonators have Bode-Q values of ~600 and ~520, respectively. The measured frequency response of the high-frequency XBAR filter is shown in Figure 7d. The center frequency is around 4.13 GHz, with a narrow passband between 4.1 and 4.2 GHz. The inserted image in Figure 7d shows the zoomed-in view of the filter near the passband. In addition to the prominent peak, a higher passing window can also be found at ~7.4 GHz, corresponding to the *A*_3_ Lamb mode. It should be emphasized that the developed prototype device is not suitable for wideband application, but it can be used as a narrowband filter to pass signals with exact frequency bands. To meet the wideband requirements, future work will focus on exploring the influence of crystal orientation and developing LT-based XBAR, enabling high *k_t_*^2^.

Meanwhile, as the resonator can operate in the super-high frequency range, it can be used for super-high frequency (SHF) low-phase-noise oscillator development. In addition, the ultra-thin LT membrane makes the XBAR susceptible to external perturbation, and it can be a competitive candidate for high-precision sensing applications.

## 5. Conclusions

In summary, the XBAR devices based on the ultra-thin (~420 nm) 42° YX-cut LT film have been demonstrated to accurately predict the performance of the resonator, a precise FEM model that was built. The simulation results showed excellent consistency with the experiment, especially when the metal loss, mechanical damping, and dielectric loss were considered. Furthermore, the temperature characteristics of the devices were thoroughly analyzed. The fabricated LT thin film-based XBARs exhibited a prominent resonance peak around 4.1 GHz, corresponding to the *A*_1_ Lamb wave. The primary resonant mode has a high Bode-Q value of ~500–800 and an effective mechanical coupling coefficient *k_t_*^2^ of ~2.84%. In addition, the *A*_0_ and *A*_3_ Lamb modes were also observed within the frequency range of 0.1~10 GHz. Moreover, a ladder-type narrowband filter comprising five XBARs has been prepared, with a center frequency of ~4.13 GHz and a 3 dB bandwidth of ~50 MHz. This work presents the precise modeling approach for the design and optimization of the piezoelectric single-crystalline thin film-based acoustics resonator. The demonstrated performance and application potential of the LT thin film-based XBAR devices open up new possibilities in the field of wireless communication and signal processing.

## Figures and Tables

**Table 1:** Properties of Cr and Au.

Material	Density (kg/m^3^)	Coefficient of Thermal Expansion (×10^−6^/°C)
Cr	7150	4.9
Au	19,300	14.2

**Table 2:** Key parameters of series and shunt.

Parameters	Λ (μm)	Aperture Witch (μm)	IDT Pairs	SiO_2_ Thickness (nm)
Series	5.9	30	50	0
Shunt	5.9	20	40	20

## Data Availability

The datasets presented in this article are not readily available because the data are part of an ongoing study. Requests to access the datasets should be directed to hexingli@suda.edu.cn.
